# Intractable Left Internal Mammary Artery Spasm After Coronary Artery Bypass Grafting

**DOI:** 10.7759/cureus.7045

**Published:** 2020-02-19

**Authors:** Vrinda Vyas, Alisha Khan, Avneet Singh

**Affiliations:** 1 Internal Medicine, State University of New York (SUNY) Upstate Medical University, Syracuse, USA; 2 Cardiology / Internal Medicine, State University of New York (SUNY) Upstate Medical University, Syracuse, USA

**Keywords:** cabg, coronary artery bypass grafting, lima spasm, graft spasm, stemi

## Abstract

Graft spasm is a rare but well-recognized complication of coronary artery bypass grafting (CABG). The occurrence of graft spam is multifactorial and can be fatal if not diagnosed and treated promptly. There are no well-defined guidelines for the management of a severe spasm. We report the case of a 29-year-old man with left internal mammary artery (LIMA) spasm in the immediate post-operative period following CABG. The intracoronary infusion of nitroglycerin relieved the spasm temporarily, confirming the diagnosis. The patient eventually underwent redo bypass grafting.

## Introduction

Persistent graft or native coronary vasospasm in the postoperative period is a potentially fatal complication of CABG. Perioperative spasm in bypass grafts and coronary arteries has been reported in 0.43% of all CABG surgeries, but this may be an underestimate. A spasm can develop not only in the internal mammary artery, but it also occurs more frequently in the right gastroepiploic and radial artery. Hence, the left internal mammary artery (LIMA) is preferred over other arteries. Severe LIMA spasm can present with angina, acute hemodynamic collapse, ST-segment elevation myocardial infarction (STEMI), ventricular fibrillation, or a combination of these. The use of calcium channel blockers, intravenous nitroglycerin, and local injections of these vasodilators, both intraoperatively during CABG and via an angiographic catheter, have been suggested to reduce the rate and severity of graft spasm.

## Case presentation

The patient is a 29-year-old male with a past medical history of poorly controlled type II diabetes mellitus, hypertension, tobacco abuse (one pack-per-day), and leukemia at the age of nine months for which he received systemic chemotherapy, total body radiation, and bone marrow transplant. The patient had not seen a doctor or been on any medications for the past five years. The patient presented to a hospital after a syncopal episode. CT head showed a right sphenoid wing meningioma, which was 4.3 x 4.1 x 2.2 cm in size with mass effect. He was admitted to the hospital and treated with hypertonic saline and levetiracetam for increased intracranial pressure.

**Figure 1:**
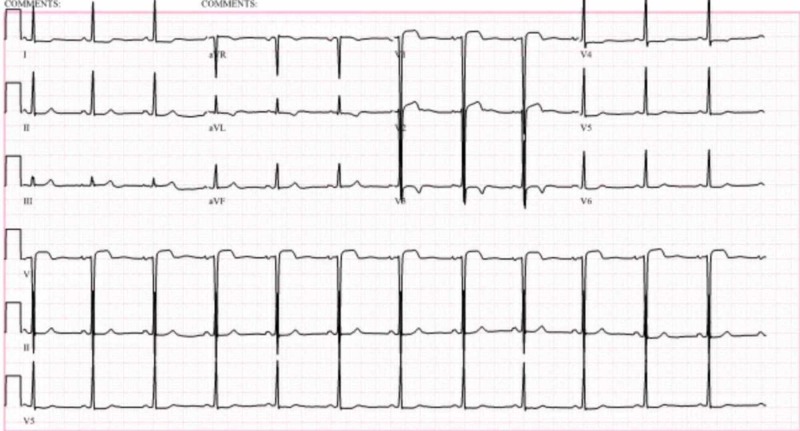
EKG on presentation to our hospital Showing moderate voltage criteria for left ventricular hypertrophy with nonspecific ST and T wave changes; otherwise normal EKG EKG: electrocardiogram

During the hospital stay, the patient reported intermittent episodes of chest pressure lasting for 15-20 minutes each. Given the typical nature of his chest pain, transthoracic echocardiography was performed, which revealed a left ventricular ejection fraction of 45%-50% with anteroseptal hypokinesis (Figure 1). He then underwent cardiac catheterization, which revealed 95% stenosis of the proximal left anterior descending (LAD), 60%-70% stenosed ostial left circumflex, and mid-portion of the right coronary artery (RCA) with 95% stenosis. It also revealed an elevated left ventricular end-diastolic pressure. The patient was then transferred to our hospital for a higher level of care.

**Figure 2:**
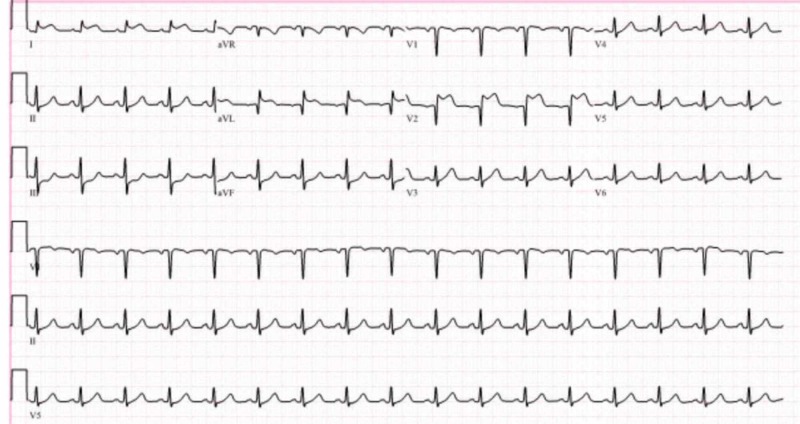
EKG within two hours of the coronary artery bypass grafting ST elevations are noted in the anterior and lateral leads with reciprocal ST depressions noted in the inferior leads, consistent with anterolateral infarct. EKG: electrocardiogram

Cardiothoracic surgery was consulted given the patient's triple vessel disease, and the patient underwent CABG with LIMA graft to the LAD and reverse saphenous vein graft to the posterolateral circumflex and posterior descending artery (PDA). Within two hours postoperatively, the patient developed ischemic changes in the anterior and lateral leads (ST elevation in V2, I, and augmented vector right (AVL)). See Figure 2.

**Figure 3:**
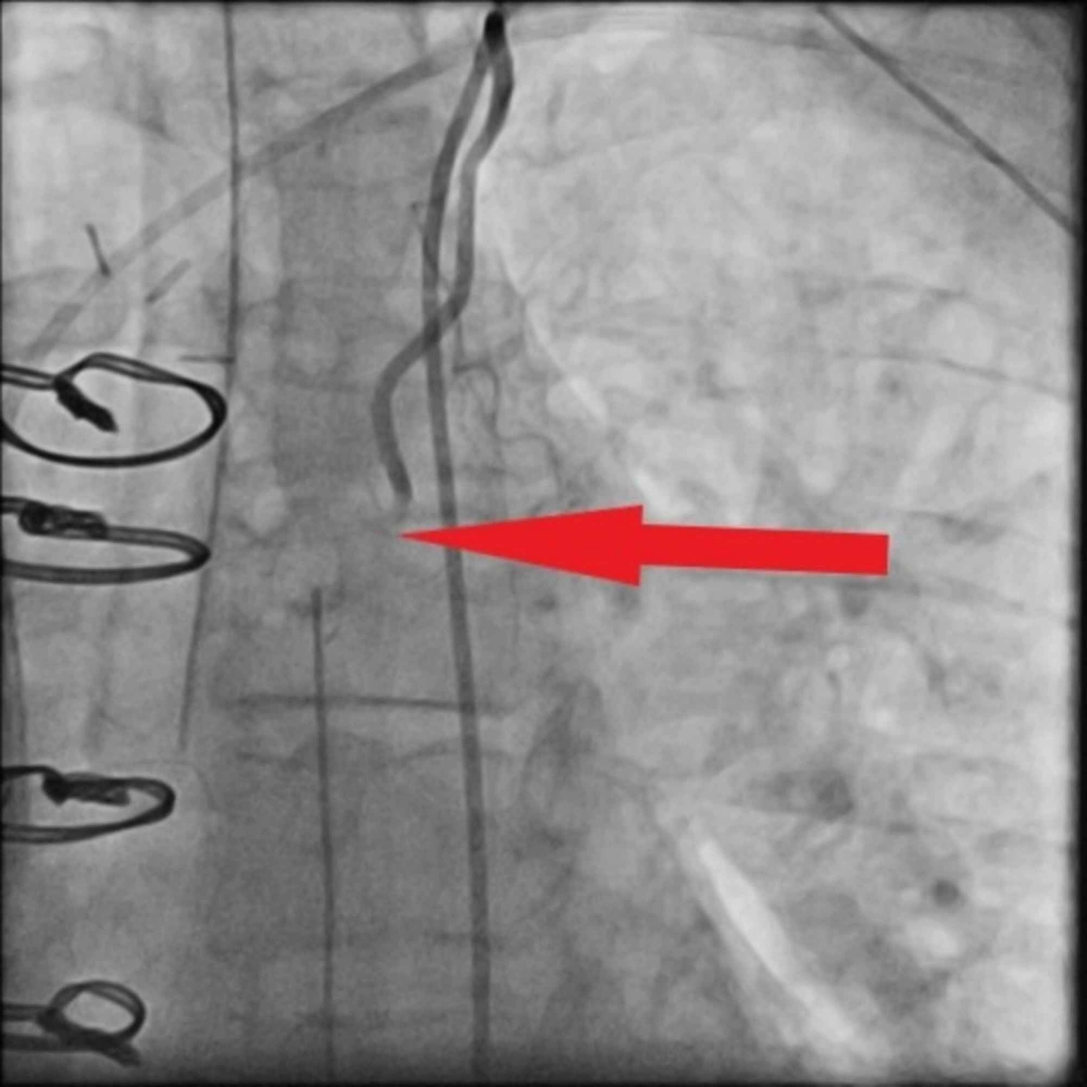
Coronary angiography images prior to the administration of nitroglycerin Arrow points toward spastic left internal mammary artery segment.

**Figure 4:**
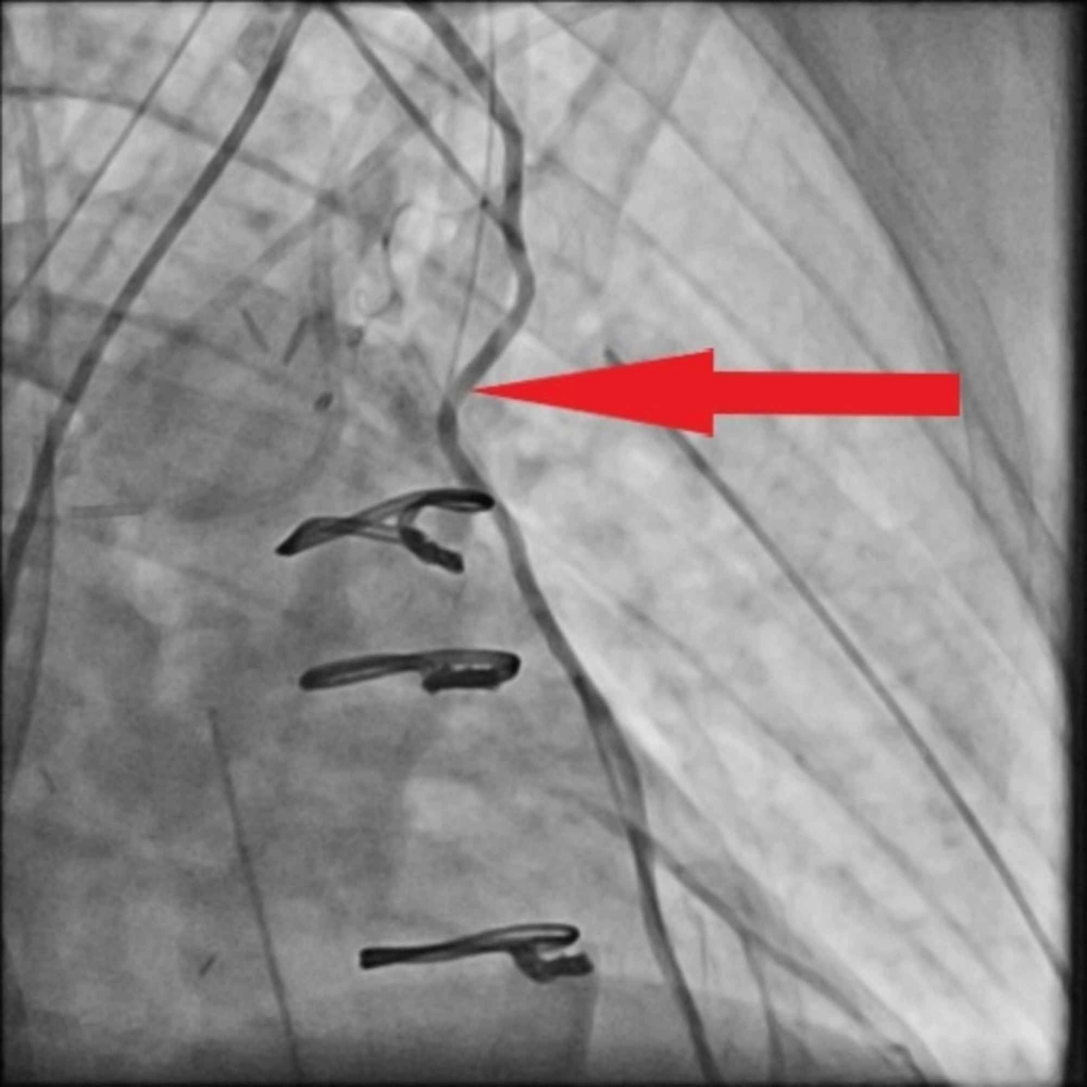
Post nitroglycerin administration, the spasm in the grafted artery is relieved.

Given the EKG findings of STEMI, the patient was taken for emergent coronary angiography, which demonstrated diffuse vasospasm in the body of the grafted LIMA conduit (Figure 3). Intracoronary infusion of nitrates successfully relieved the spasm (Figure 4). The patient was then continued on a low-dose nitroglycerin infusion for a day after the angiography.

On the first postoperative day, the patient again experienced angina and persistent ST changes were noted in leads V2, V3, and V4 with a maximum troponin level of 3.46 ng/ml. He underwent a redo CABG emergently with anastomosis of the reverse saphenous vein graft to the distal LAD and aorta. The patient’s recovery was uneventful thereafter, and he was discharged home in a stable condition on the ninth postoperative day after the redo CABG.

## Discussion

CABG remains the standard of treatment in patients with multivessel coronary artery disease. Using autologous arteries as bypass grafts has been proven to provide superior outcomes when compared to venous grafts. The use of LIMA to graft the diseased LAD has become the standard of practice in CABG because LIMA is generally free of atherosclerosis and has high patency rates as grafts [1-2]. Except for the internal mammary artery (IMA), other arterial grafts (such as the radial artery, gastroepiploic artery, inferior epigastric artery, and so on) have a greater muscular composition in the vessel wall and a consequent tendency to develop vasospasm during surgery. Therefore, antispasmodic therapy remains an important component of CABG.

The first report of early postoperative spasm was reported in a LIMA graft in 1987 [3]. Subsequently, it was reported that spasm could be a localized phenomenon or a diffuse process, referred to as a “string sign.” Clinically, spasm of the IMA usually causes ischemia typically occurring immediately after the discontinuation of cardiopulmonary bypass during the CABG but has also been seen subsequently in the first few postoperative hours, when it may also be confused with native coronary artery spasm [4]. Spasm of the LIMA has also been documented years after CABG, although that is even rarer [5].

Perioperative spasm in bypass grafts and coronary arteries has been reported in 0.43% of all CABG surgeries and the incidence of refractory and lethal spasm is at least 0.12 %, but this may be an underestimate, as, often, spasm may be transient and asymptomatic [6]. The development of spasm is often sudden, and angiography may characteristically show diffuse arterial spasm of LIMA grafts as well as the native coronary arteries [7-9].

The exact mechanism of graft and/or native coronary spasm in the perioperative period is still unclear, but an interplay of several factors is thought to precipitate vasospasm. These factors include damage to the vessel at the time of dissection, stimulation of alpha-receptors by vasoconstricting mediators and inotropes, alkalosis, and abrupt cessation of vasodilators used during the operation [3,10]. Because of the complexity of the pathophysiological processes causing conduit spasm, there is no single technique or drug that has been universally accepted as the treatment of choice to prevent or treat graft spasm.

LIMA spasm can present as acute hemodynamic collapse, ST-segment elevation, ventricular fibrillation, or a combination of these. When spasm happens in the late postoperative period after a CABG when the patient has been extubated, the patient may complain of chest pain, back pain, sudden respiratory distress, diaphoresis, and a sudden drop in blood pressure and heart rate, with ST-segment changes, may be noted [11].

All patients suspected of having graft spasm should be transferred immediately to the cardiac catheterization laboratory for coronary angiography [12]. When the diagnosis of graft spasm is confirmed, intraluminal vasodilator injections (such as nitroglycerin, verapamil, diltiazem) via angiographic catheters may rapidly relieve the spasm [8-9]. Rarely, coronary angioplasty and stenting of the graft have also been tried to treat life-threatening postoperative vasospasm, which did not resolve with conventional vasodilator therapy [13].

Intraaortic balloon pump (IABP) support is often necessary when the patient’s hemodynamics are unstable [9]. Rarely, extracorporeal membrane oxygenation (ECMO) has been used to help with the hemodynamic compromise because of severe intractable LIMA spasm [9]. Intractable graft spasm is potentially lethal by causing myocardial infarction and progressing to develop fatal ventricular fibrillation and cardiac arrest. In one study, five out of seven patients with refractory spasm rapidly deteriorated and developed catastrophic ventricular fibrillation and cardiac arrest [9]. Hence, it is extremely crucial to make a prompt diagnosis and immediately treat these patients with intraarterial injections of vasodilators.

Our patient was started on a nitroglycerin infusion after confirming the diagnosis of LIMA graft spasm, but he continued to experience signs and symptoms of ongoing myocardial infarction correlating with the region of the LIMA graft, necessitating a second CABG wherein the LIMA graft to LAD was bypassed with a reverse saphenous venous graft, following which the patient’s symptoms ultimately subsided. We have described this case to emphasize the importance of timely diagnosis and an aggressive approach for the management of this rare and the potentially lethal complication of CABG surgery.

## Conclusions

Spasm of the graft vessel in the postoperative period following a CABG is a multifactorial process that is difficult to predict. Graft vasospasm is usually treated with an injection of intraluminal vasodilators such as nitroglycerin, diltiazem, verapamil, and papaverine. Intractable graft spasm, which does not respond to vasodilator therapy, can lead to STEMI and can be complicated by hemodynamic collapse and ventricular arrhythmias. Coronary artery angioplasty and stenting of the graft have been used to treat intractable graft vessel spasm but there is no consensus on treatment, and our patient eventually required a redo CABG to relieve the ongoing LIMA graft spasm. Our case illustrates the life-saving potential of coronary artery angiography to establish an early diagnosis of this rare but potentially fatal complication of CABG.
